# Editorial: Hormone Receptors and Breast Cancer

**DOI:** 10.3389/fendo.2019.00205

**Published:** 2019-04-02

**Authors:** Antonio Brunetti, Guidalberto Manfioletti

**Affiliations:** ^1^Department of Health Sciences, University “Magna Graecia” of Catanzaro, Catanzaro, Italy; ^2^Department of Life Sciences, University of Trieste, Trieste, Italy

**Keywords:** estrogen receptors, steroid receptors, tyrosine kinase receptors, hormone resistance, triple negative breast cancer

Breast cancer (BC) is the most commonly diagnosed cancer among women worldwide and one of the leading cause of cancer-related deaths (1). The majority of BCs arise from epithelial cells, either in the ducts or lobules, as the result of genetic and epigenetic alterations, which lead to aberrant growth control and disruption of intracellular signaling. Because of this, BC is considered a heterogeneous disease with multiple sub-types, with cells of distinct origin and function (2, 3).

Overexpression of estrogen receptors (ERs), during BC development, has a significant impact, since ~75% of BCs expresses ERs (4). ERs are transcription factors that, upon binding to the estrogen steroid hormone, migrate to the nucleus, driving specific gene expression programs that intercept growth control pathways. Therefore, inhibiting ER, particularly ERalpha, through the use of hormone-based therapeutics such as tamoxifen, fulvestrant and aromatase inhibitors, is a primary goal of endocrine therapy in BC (5). The widespread use of these drugs as adjuvant therapies for BC has led to a significant reduction in mortality, although intrinsic and acquired resistance to these inhibitors may limit their efficacy. In this regard, ER mutations or loss of expression, alteration in ER coactivators/corepressors, overexpression and/or amplification of growth factor receptors which impinge on the PI3K/AKT/mTOR and RAF/MEK/ERK pathways, and alterations of cell-cycle checkpoints confer resistance to endocrine therapy (6). In recent years, our understanding of the mechanisms underlying hormone-therapy resistance in BC has improved, and this has led to the approval of three anticancer agents: the mTOR inhibitor everolimus, and the two CDK 4/6 inhibitors, palbociclib, and ribociclib (7). However, despite these successful advances, novel targets need to be identified, together with reliable and reproducible biomarkers of BC.

Apart from the intrinsic or acquired hormone-therapy resistance, there is a consistent number of breast tumors that do not express ERs. BCs lacking ERalpha and progesterone receptors, along with no overexpression of epidermal growth factor receptor 2, constitute the so-called triple-negative BC (TNBC) (8), an aggressive BC subtype that comprises 10–30% of all BCs and is associated with younger age and poorer prognosis (9). TNBCs are a group of heterogeneous BCs and represent a challenge for therapy. On the other hand, other steroid receptors, such as vitamin D receptor (10), glucocorticoid and androgen receptors (11, 12), as well as tyrosine kinase receptors, including IGF-1 and insulin receptors (13–15), can be also expressed in breast cells, and their roles and significance in cancer cell growth might deserve further investigation.

The aim of this Research Topic was to gather up-to-date data and novel point of view in the field of hormone receptors and BC.

In addition to the classical actions of E_2_-ERalpha on gene transcription, E_2_-ERalpha exerts extranuclear actions that are important for activating signal transduction pathways. Among these actions, activation of the anticipatory Unfolded Protein Response (UPR) is the most recently described. Livezey et al. reviewed the role of mitogenic hormones in activating the UPR, an endoplasmic reticulum stress response pathway, in anticipation of a future need for increased protein folding capacity upon cell proliferation. Overexpression of UPR components strongly correlated with reduced time to recurrence, subsequent resistance to tamoxifen, and reduced survival in ERalpha-positive BC. Because of this, UPR components are considered promising targets in ERalpha-positive cancers and, in this regard, several drugs are currently being studied. Among these drug molecules, the Authors focused on a promising preclinical drug candidate, BHPI, that selectively targets ERalpha-positive cancer cells, blocking proliferation and killing tumor cells. Hence, Authors suggest exploiting the UPR pathway as a target for the development of novel anticancer drugs.

Sanchez et al. contributed a research article in which they experimentally demonstrated that gonadotrophins FSH/LH can alter the expression of genes involved in cell adhesion, motility, and invasion via activation of their own receptors in human BC cells. This activity is mediated by moesin and FAK kinases, two actin cytoskeleton regulator proteins, whose expression and activation are modulated by FSH/LH. In a rat model of BC progression, Authors provide evidence for a direct correlation between gonadotropin levels and cancer growth. Based on this, they suggest that gonadotropins may thus influence BC progression, particularly in post-menopausal women with higher FSH/LH levels.

Bond et al. provided an overview of the functional role of ZNF423 in human malignancies, with a particular focus on its implication in hormone-responsive BC. As a zinc-finger protein, ZNF423 interacts with different activators, inhibitors, and co-interacting transcription factors, playing a key role in development and disease, including cancer. SNPs in the *ZNF423* gene, which were associated with decreased risk of BC during selective ER modulators therapy, have been identified by GWAS, providing an opportunity for the identification and stratification of patients that are more likely to respond to this treatment. A role for ZNF423 in the control of the estrogen response was supported by findings indicating that ZNF423 expression can be induced by estrogens through specific response elements within the *ZNF423* gene, which in turn promotes BRCA1 expression, thus modulating the DNA damage response. In line with this is the finding that ZNF423 knocked-down cells were more sensitive to the PARP inhibitors olaparib and cisplatin, consistent with a BRCA1 decrease. In the Author's opinion future studies may establish if ZNF423 is of clinical benefit.

Cusan et al. reviewed the mutational spectrum of the *CDKN1B* gene that encodes for the p27^Kip1^ protein, a key regulator of cell-cycle. Although firstly characterized as a cyclin dependent kinase-inhibitor, many studies have demonstrated that p27^Kip1^ can accomplish several non-canonical functions, being able to interact with many different proteins having a central role in tumor progression (16). p27^Kip1^ is mainly regulated at post-transcriptional level; however, massive parallel sequencing demonstrated that, in some types of cancer, including BC, *CDKN1B* is mutated at relatively high frequency (17). By reviewing all *CDKN1B* mutations in cancer, the Authors highlight that mutations affecting the *CDKN1B* gene are present and can represent driver genetic lesions in the ER-positive luminal BC subtype. They emphasize that more work is necessary to fully clarify the role of p27^Kip1^ in BC and other hormone-driven tumors.

In summary, these articles contribute to understanding the topic and gathering information about the molecular mechanisms that are involved in BC development and progression. A better knowledge of the mechanisms involved in the pathogenesis of BC could enable us to discovery new biomarkers for patient stratification and identify novel therapeutic targets.

## Author Contributions

All authors listed have made a substantial, direct and intellectual contribution to the work, and approved it for publication.

### Conflict of Interest Statement

The authors declare that the research was conducted in the absence of any commercial or financial relationships that could be construed as a potential conflict of interest.
